# The Endophytic *Pseudomonas* sp. S57 for Plant-Growth Promotion and the Biocontrol of Phytopathogenic Fungi and Nematodes

**DOI:** 10.3390/plants10081531

**Published:** 2021-07-27

**Authors:** Patricio Muñoz Torres, Steffany Cárdenas, Mabel Arismendi Macuer, Nelly Huanacuni, Wilson Huanca-Mamani, Denise Cifuentes, Germán F. Sepúlveda Chavera

**Affiliations:** 1 Laboratorio de Patología Vegetal y Bioproductos, Facultad de Ciencias Agronómicas, Universidad de Tarapacá, Av. General Velásquez 1775, Arica 1000000, Chile; sfcninasivincha@gmail.com (S.C.); arismendimabel@gmail.com (M.A.M.); nelly.huanacuni@gmail.com (N.H.); whuanca@uta.cl (W.H.-M.); 2 University of California Davis Chile Life Sciences Innovation Center, Av. Santa María 2670, Santiago 7520424, Chile; dcifuentes@ucdavis.edu

**Keywords:** Atacama Desert, endophyte, plant-growth promoting bacteria, biocontrol, pre-Andean agriculture

## Abstract

Oregano from Socoroma (Atacama Desert) is characterized by its unique organoleptic properties and distinctive flavor and it is produced using ancestral pesticide-free agricultural practices performed by the Aymara communities. The cultivation in this zone is carried out under extreme conditions where the standard production of different crops is limited by several environmental factors, including aridity, high concentration of salts, and boron among others. However, oregano plants are associated with microorganisms that mitigate biotic and abiotic stresses present in this site. In this work, the S57 strain (member of the *Pseudomonas* genus that is closely related to *Pseudomonas lini*) was isolated from roots of oregano plants, which are grown in soils with high content of non-sodium salts and aluminum. This bacterium stimulates the growth of Micro-Tom tomato plants irrigated with saline-boric water. Moreover, it controls the growth of phytopathogenic fungi *Fusarium oxysporum* and *Botrytis* *cinerea* and the nematode *Meloidogyne incognita* under saline-boric conditions. Together with the high levels of bacterial biomass (~47 g/L), these results allow the establishment of the bases for developing a potential new agricultural bioproduct useful for arid and semiarid environments where commercial biological products show erratic behavior.

## 1. Introduction

Socoroma (in Aymara *Chukuruma*; running water) Valley (18°15′37″ S, 69°36′24″ W) is located at the Andean pre-mountain range of the Arica and the Parinacota Region at 3070 m.a.s.l. in the Commune of Putre in the extreme north of the Atacama Desert, Chile [1]. This valley is characterized by the cultivation of oregano [2], which possesses unique organoleptic properties, possesses geographical indication distinction and granted the seal of origin by the National Institute of Intellectual Property, INAPI, Chile [3]. Particularly, oregano cultivation is performed under ancestral agricultural practices, in which crops are maintained employing pesticide-free applications, harboring unexplored microbial genetics resources associated with this type of plants. Microbial communities associated to plant tissues (plant microbiome) are considered beneficial because they contribute to the plant by mitigating biotic and abiotic stress conditions [4,5]. Thus, this allow plants to survive under the extreme conditions that prevail in this region, including aridity, high concentrations of salts and boron, a wide thermal amplitude between the day and night, poor soil composition, and high UV radiation among other extreme environmental conditions which are restrictive for agricultural activities in arid and semiarid regions [6,7].

Under the extreme conditions of this zone, plant-associated bacteria play a crucial role in maintaining the proper functioning of plants [8]. These bacteria are known as plant-growth-promoting rhizobacteria (PGPR) and correspond to a free-living soil bacteria with the ability to colonize the rhizosphere and roots, which promotes the growth through the release of metabolites that act directly in plants [9] including the production of phytohormones, such as indole-3-acetic acid (IAA), gibberelins, and cytokinins [10,11]; and the fixation of elemental nitrogen [12]. Furthermore, the solubilization of minerals also occur, such as inorganic phosphate and iron [13,14], and the biological control of phytopathogenic organisms through the production of inhibitory compounds and hydrolytic enzymes also occur [15].

Beneficial PGPR associated with oregano plants from Socoroma could increase interest due to the extreme conditions where they are cultivated. Under this type of conditions, bacteria could confer tolerance to the different stresses to which oregano crops are naturally subjected to, which allows plant survival [11]. Although different bacteria have been isolated from several plants [16], PGPR associated with oregano from Socoroma have been scarcely described and harbors a high potential to characterize the plant-microorganism interaction under extreme conditions of cultivation, to describe adaptation mechanisms to this kind of environment, and to exploit new natural compounds for biotechnological and agricultural purposes. This study was focused on the isolation, identification, and in vitro and in planta functional characterization of the *Pseudomonas* sp. strain S57, which was obtained from oregano roots from Socoroma. The S57 isolate was selected from the plant-associated bacterial culture collection of the Laboratory of Plant Pathology and Bioproducts (Universidad de Tarapacá, Chile) because it possesses promissory plant-growth promoting activities making it an excellent candidate for the development of a new bioproduct. This strain is closely related to *Pseudomonas lini* and qualitative functional analysis revealed that this bacterium possesses plant-growth-promoting activities and a wide range of antifungal properties against several phytopathogenic fungi, including under saline-boric conditions. Furthermore, bacterial cultures were optimized in a flask and bioreactor to produce high quantities of biomass to perform the experimental procedure in Micro-Tom tomato plants and not only to establish the baseline for the development of a new bioproduct tolerant relative to the extreme conditions present in the valley of Arica and Parinacota Region, Chile, but also relative to arid and semiarid environments.

## 2. Results

### 2.1. Soil Samples Characterization

Three samples were taken from different sites in Socoroma to characterize arable soils and to establish the extreme conditions where oregano plants are cultivated (Table 1). The S1 sample showed high levels of total nitrogen, phosphorous, potassium, boron, aluminum, and copper and accepted values of sulfur and sodium were found. For the S2 sample, excessive levels of total nitrogen, phosphorous, potassium, sulfur, aluminum, and copper were detected. Meanwhile, boron and sodium levels were in accepted values. S3 sample showed high levels of potassium and aluminum; deficient levels of total nitrogen, sulfur, and boron; and accepted values for phosphorous, copper, and sodium. The NPKS content in the three samples showed a direct relationship with the organic matter content. All the samples showed high electrical conductivity, suggesting non-sodium highly saline soils due to the low concentration of sodium. Measured pH values indicated the presence of strong acid soils for S1 and S2 samples and slightly acid soil for S3.

Pesticide residues analysis (not showed) included the detection of 163 insecticides, acaricides, and nematicides; 74 herbicides and plant growth regulators; and 93 fungicides. No pesticide residues were detected in the three samples, which confirms free-pesticides agricultural practices.

### 2.2. S57 Strain Characterization and Identification

The S57 strain was isolated as an endophytic bacterium from roots of oregano plants from Socoroma. Microbiological characterization (Table 2) showed Gram negative straight rod cells, which are motile in semi-solid media and able to produce gas after the addition of 3% H_2_O_2_, which indicates the production of catalase enzyme. The colonies were yellowish, circular, and convex with regular margins after 2 days of growth on King’s medium B agar. This bacterium produced acids from D-mannitol, D-glucose, D-fructose, D-galactose, D-rhamnose, D-melibiose, sucrose, and lactose, but no acid production was detected when D-sorbitol and D-cellobiose were used as carbon sources.

Antibiotic susceptibility of the S57 strain was performed by the disk diffusion method (Table 3), observing that this bacterium was sensitive to chloramphenicol (50 μg), ciprofloxacin (1 and 10 μg), and kanamycin (30 μg). S57 showed intermediate resistance to chloramphenicol (10 μg), kanamycin (5 μg), and neomycin (10 and 30 μg). Differences in the susceptibility using chloramphenicol and kanamycin could be explained by a lesser diffusion of the antibiotic subjected to compound concentration. Moreover, this strain was resistant to amoxycillin, ampicillin, and penicillin G, which is expected due to β-lactam antibiotics being mainly active against Gram positive bacteria.

**Figure 1:**
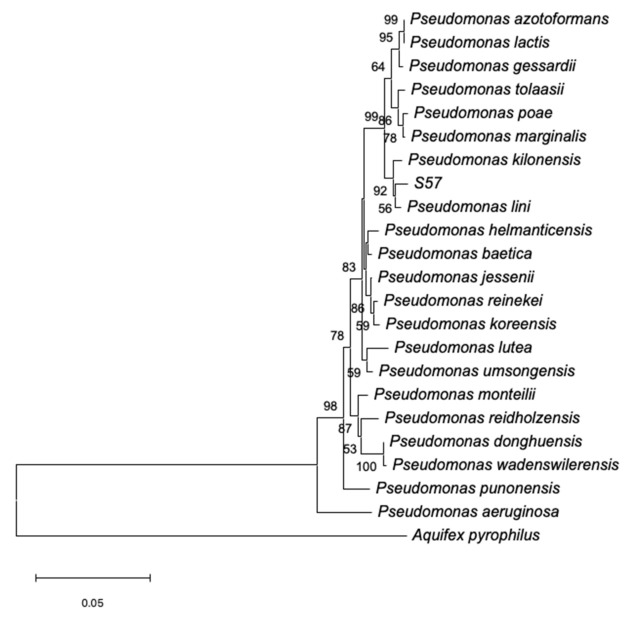
Neighbor-joining tree of the partial 16S rRNA gene sequence of the S57 strain and the closely related species of *Pseudomonas* genus.

Sequencing and comparison of 16S rRNA gene to sequences deposited in GenBank revealed that the S57 strain is a member of *Pseudomonas* genus and the phylogenetic analysis using the Neighbor-Joining method (Figure 1) showed the S57 strain was closely related to the PGPR *Pseudomonas lini*.

### 2.3. Tolerance to NaCl and H_3_BO_3_ of the S57 Strain

A qualitative methodology was performed to determine the tolerance of the S57 strain to saline-boric conditions (Table 4). Abundant growth was observed in King’s medium B supplemented with 8 g/L and 15 g/L of NaCl or 10–100 ppm of H_3_BO_3_ similar to the control conditions in the absence of NaCl and H_3_BO_3_. However, poor growth was detected when 20 g/L NaCl was added to the medium. When the liquid medium was supplement with 0.86 g/L NaCl and 114 ppm of H_3_BO_3_ (1× Lluta irrigation water), abundant growth of the S57 strain was detected. Furthermore, poor growth was observed using 9× Lluta irrigation water and no growth was perceived using higher concentrations of the NaCl- H_3_BO_3_ mixture.

### 2.4. In Vitro PGP Traits and Antifungal Activity of the S57 Strain

In vitro PGP activities of the S57 strain are described in Table 5. S57 bacterium grew in NFb semisolid medium and formed a subsurface veil-like pellicle indicating the bacterial ability to fix elemental nitrogen. Furthermore, the S57 strain was capable of solubilizing phosphate in PVK solid medium as it was observed through the apparition of a clear halo rounding the colony. After 5 days of inoculation in fresh King’s medium B, the S57 isolate was able to produce 7.9 μg/mL of IAA and 24.2 psu of siderophores.

The antifungal activity of the S57 strain is shown in Table 6. Under standard conditions (absence of NaCl and H_3_BO_3_), S57 isolate showed antifungal activity against the phytopathogens *Fusarium oxysporum* (23.5%), *Botrytis cinerea* (48.3%), *Geotrichum candidum* (58.6%), and *Monilinia fructicola* (67.2%), reaching IMRG percentages ≥ 50% for the last three fungi. When 10 g/L NaCl and 110 ppm H_3_BO_3_ were added to the culture medium (saline-boric conditions), antifungal activities against *F. oxysporum* and *B. cinerea* were unaltered; meanwhile, the ability to control *G. candidum* and *M. fructicola* decreased to 29.6% and 52.3%, respectively. Due to *F. oxysporum* and *B. cinerea* being the most common phytopathogenic fungi present in the Arica and Parinacota Region, a dual culture assay using irrigation water from the Lluta River instead of distilled water was performed and observed that the use of Lluta irrigation water did not affect the antifungal activity against these phytopathogenic fungi.

### 2.5. Growth Optimization of the S57 Strain in Flask and Bioreactor Conditions

Three parameters (temperature, pH, and agitation) were modified independently to optimize the bacterial growth of the S57 strain (Table 7). No significant differences were observed in the generational time and the microbial growth rate when incubation temperature was varied from 25 °C to 35 °C, as is observed in Table 7; 35 °C being the temperature with the highest μ (2.98 h^−1^) and the lowest g (0.33 h) and it is considered as the optimum growth temperature. A significant decrease in μ and increase in g were registered at higher temperatures.

Higher microbial growth rates and lower generational times were determined when the S57 isolate was inoculated in a buffered culture medium in the pH range from 5.5 to 7.5 (Table 7). When King’s medium B was buffered at pH 5.0, a decrease in μ (2.07 h^−1^) was observed. The pH range between 5.5 and 6.0 was considered as optimum pH for bacterial growth in the bioreactor.

The lowest generational time (0.33 h) and the highest microbial growth rate (3.06 h^−1^) were registered when the S57 strain was grown using 150 rpm of agitation (Table 7) and this is considered as the optimum agitation speed. Higher g values and lower μ rates were determined when the agitation speed is reduced.

Optimization in the bioreactor was performed using a two-variables experiment (Table 13), where aeration and impeller speed (agitation) were modified simultaneously to increase the cellular mass of the S57 strain. Optimal temperature and pH were selected from in flask assays. Two-variables experiment results are shown in Table 8. Higher CFU/mL (2.5 × 10^27^ CFU/mL) and biomass (47.1 g/L) were obtained when experiment number six was carried out (0.5 VVM of aeration and 75 rpm of agitation) and its parameters were considered as optimal parameters for the growth of the S57 strain in the bioreactor. However, no bacterial growth was observed when 150 rpm of impeller speed was used after 48 h post-inoculation (not showed).

### 2.6. Effect of the Temperature and the Addition of Stabilizing Agents to Formulations of the S57 Strain

**Figure 2:**
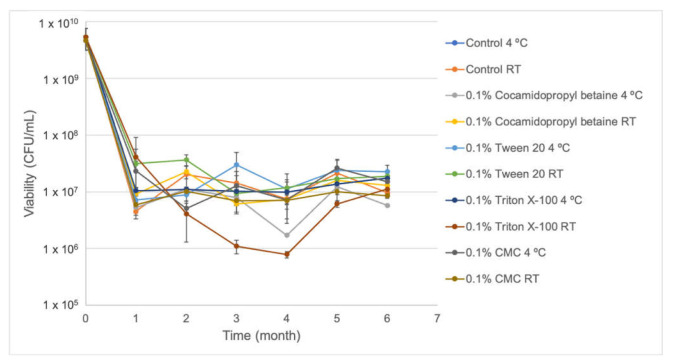
Viability of five formulations of the S57 strain stored at room temperature and 4 °C. Error bars represent the standard deviation of the independent assays.

Viability of the S57 strain in liquid culture medium in the presence and absence of stabilizing agents carboxymethylcellulose (CMC), cocamidopropyl betaine, Tween 20, and Triton X-100 at 0.1% final concentration was evaluated for 6 months when a dilution of ~5 × 10^9^ CFU/mL was prepared and stored at room temperature and 4 °C (Figure 2). It was observed that viable bacterial counting decreased over the month for the five formulations stored at room temperature and 4 °C. The formulations reached values of ~1 × 10^7^ CFU/mL after one month of storage at room temperature and kept constant for 6 months, except for 0.1% Triton X-100 for which viability was reduced to ~1 × 10^6^ CFU/mL up to the fourth month. After this, the S57 viability was recovered reaching values similar to the other formulations. Similar behavior was observed at 4 °C where the viable count diminished to values close to 1 × 10^7^ CFU/mL after six months of storage, except for 0.1% cocamidopropyl betaine stored at 4 °C, which possesses the lowest viability after six months (5.73 × 10^6^ CFU/mL).

**Figure 3:**
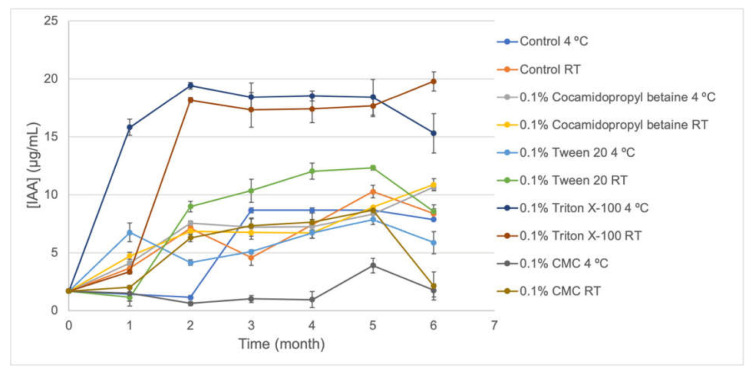
IAA production of five formulations of the S57 strain stored at room temperature and 4 °C. Error bars represent the standard deviation of the independent assays.

Auxin production by the S57 strain was variable according to the formulation employed (Figure 3). The highest values were obtained for formulations containing 0.1% of Triton X-100 stored at room temperature and 4 °C for six months and values reached 19.78 μg/mL and 15.31 μg/mL, respectively. These results suggest that this formulation produces a biostimulant based on the S57 bacteria and 0.1% of Triton X-100. Meanwhile, the lowest values were obtained for 0.1% of CMC after six months of storage in which IAA productions were 1.72 μg/mL (4 °C) and 2.10 μg/mL (room temperature).

**Figure 4:**
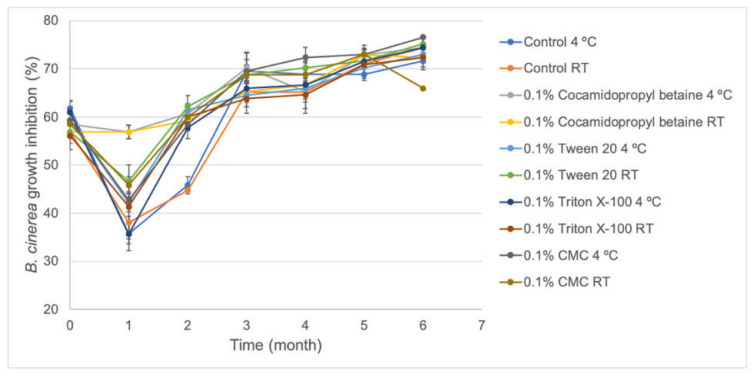
Antagonistic activity against *B. cinerea* of five formulations of the S57 strain stored at room temperature and 4 °C. Error bars represent the standard deviation of the independent assays.

Antagonistic activity against *B. cinerea* by the different formulations of the S57 strain was evaluated for six months to determine if this activity could be reduced due to storage (Figure 4). It was possible to observe that four formulations (Control, Tween 20, Triton X-100, and CMC) reduced the biocontrol activity against *B. cinerea* considerably during the first month, while cocamidopropyl betaine formulations did not show important losses for this activity. Furthermore, all formulation stored at the two temperatures exhibited a considerable increase in antagonistic activity in vitro and reached values ~75% of mycelial growth inhibition of *B. cinerea*, except for the 0.1% CMC formulation stored at room temperature in which mycelial growth inhibition reached ~66%. These results reveal that all formulations increased the antagonistic activity of S57 against *B. cinerea* during storage.

### 2.7. PGP Activity of the S57 Strain in Micro-Tom Tomato Plants

**Figure 5:**
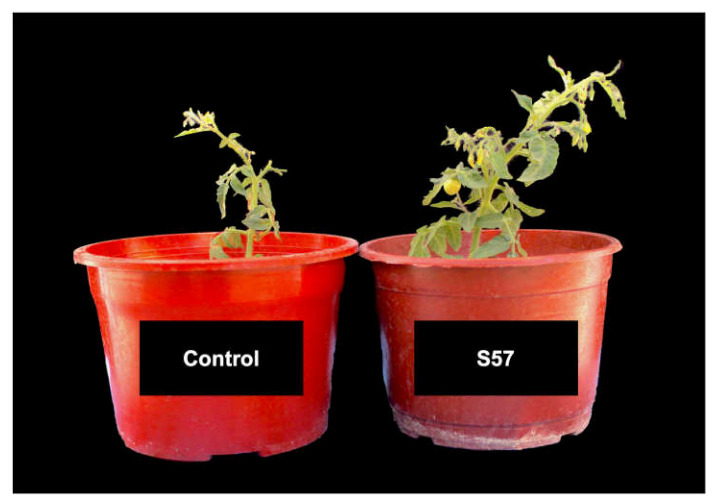
Representative image of Micro-Tom tomato plant growth treated with the S57 strain in comparison to untreated plants (control). Plants were irrigated with saline-boric water from Azapa Valley and grown in perlite as substrate.

In order to test the plant-growth promotion ability of the S57 strain, Micro-Tom tomato plants were inoculated with fresh 1 × 10^8^ CFU of this bacterium (S57 treatment) and compared with uninoculated plants or control conditions (Table 9 and Figure 5). A higher development in the aerial part of the plant was measured when Micro-Tom tomato plants were treated with the S57 bacterium and an increase of 2.27-fold was observed in the wet weight and 1.33-fold in stem length compared to uninoculated plants. However, no significant differences were observed in the dry and wet weight of treated roots compared to the control.

### 2.8. Biocontrol Aactivity of S57 Strain in Plants Against Phytopathogenic Fungi

Table 10 shows the height of bell pepper plants after 30 days of cultivation. Considering the conditions of the evaluation, plants exposed to *B. cinerea* and *F. oxysporum,* statistical analysis showed significant differences between the treatments (*p* ≤ 0.05; capital and lowercase letters, respectively), which allowed two level grouping: Control and Serenade (Gold Standard, GS) as statistical group one; and S57 as a statistical group two. For bell peppers plants in the presence of *B. cinerea*, the S57 treatment was higher than control and the GS; meanwhile, in the presence of *F. oxysporum*, S57 and GS treatments were equal.

Fresh weight was determined from the third leaf in bell pepper plants cv. Almuden (Table 11) after 30 days of cultivation in pots to determine the effect of the S57 treatment in the plant growth. For this evaluation, a random design was considered with four replicates; this allowed the definition of two statical groups: GS with lower weight; and S57 with the same weight as the control and grouped in a second superior level.

**Figure 6:**
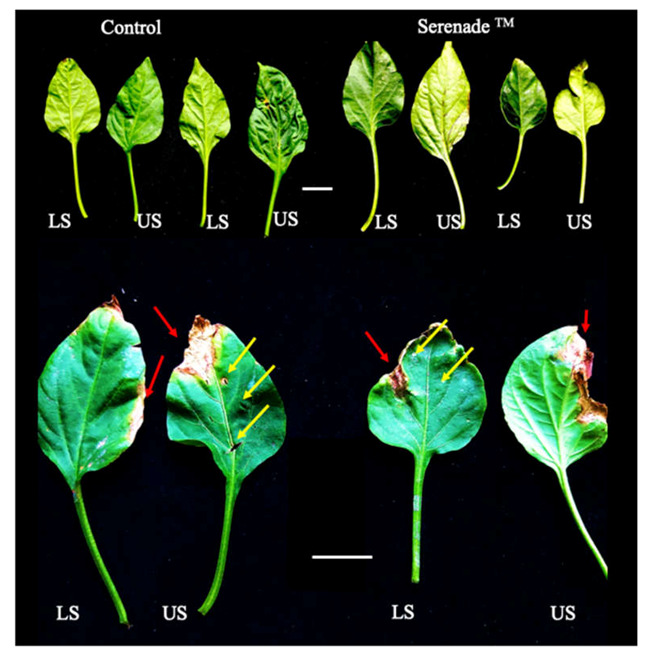
Development of the third leaf in bell pepper plants cv. Almuden after undergoing 5 treatments. Red arrows indicate *B. cinerea* damage points. Yellow arrows indicate inoculation wounds without infection. Abbreviations: LS: lower side; US: upper side. White scale bars represent 2 cm.

When evaluating the effect of the treatments on *B. cinerea* damage (Figure 6), no significant differences were detected between the treatments even though they effectively exert a mitigating effect equal to the GS. Despite this, the infection was limited in all treatments and the foliar development of the treatments was adequate according to the plant phenological state (Figure 5).

### 2.9. Nematicide Activity of S57 Strain in Plants Against Meloidogyne Incognita

As expected, the non-inoculated tomato plants cv. *Poncho Negro* did not present galls, while the plants with *M. incognita* inoculum (without any controller) presented 2.428 galls/100 g of roots. The plants treated with fluopyram presented 2 galls/100 g of roots (Table 12) and presented data that validates the extensive use of this nematicide in commercial productions. From the treatment with the S57 strain, it was possible to count 689 galls/100 g of roots. This information allows consideration that the S57 bacterium exerts a moderate effect on the root-knot formation of *M. incognita* in tomato plants. By using 4–5 separate applications every 5 to 10 days, the inhibitory effect on the formation of galls could probably be higher.

## 3. Discussion

Arid and semi-arid environments are widely distributed worldwide, occupying between 30% to 40% of the global terrestrial surface. Under these conditions, soil fertility is constrained by different environmental factors, including wide thermal amplitudes, low water availability, reduced bioavailable nitrogen and phosphorous, decreased water-holding capacity, and extreme pH values, low soil organic matter (ranging from 0.1% to 3%) among other specific limitations [17]. Socoroma soils have been maintained using ancestral agricultural practices and pesticides-free soil managements, which was confirmed by pesticides residues analysis for 330 types of agrochemicals (not showed). However, several plant limiting factors were detected (Table 1), including increased values in electrical conductivity higher than 80 dS/cm with a reduced quantity of Na (less than 0.33 cmol/kg), suggesting non-sodium highly saline soils. Salinity affects the productivity and yields of crops by reducing plant biomass, leaf area, and growth, which can be explained by the increase in soil osmotic pressure. The interference in the nutrient and water uptake; diminished CO_2_ availability with reduced photosynthetic pigments content that directly affects photosynthesis; and salt accumulation in roots that promotes the development of osmotic stress and disrupt cell ion homeostasis by inhibiting the uptake of essential elements (K^+^, Ca^2+^, and NO_3_^−^) and favors the accumulation of Na^+^ and Cl^−^ and promoting specific ion toxicities, which causes the inhibition of photosynthesis and protein synthesis, inactivate enzymes, and damages chloroplasts and other organelles [18].

Furthermore, high aluminum content was detected in Socoroma soils, which can exert a toxic effect on plants and diminishes their growth. As the concentration of aluminum increases, the soil pH decreases and the toxic effect of this metal appears, which have an important growth-limiting factor in acid soils below pH 5.0 (as it was observed in S1 and S2 soil samples, Table 1) where Al^3+^ ions predominate. Under these conditions, aluminum interferes with the elongation of root tips and lateral roots. Cell walls become more rigid by the formation of cross-linking pectins; inhibits DNA replication through the increase in DNA rigidity; bonds to phosphorous forming less available and insoluble compounds in soil and on root surfaces and thereby creating phosphorous deficiency; and affects the metabolism, disturbs the essential nutrient uptake and transport, and causes alteration in nutrient balance [19,20].

Plants possess mechanisms to reduce the toxic effect mediated by salinity and aluminum. In addition, a significant contribution to mitigating a variety of abiotic stress is carried out by plant-associated microorganisms at the plant phyllosphere, rhizosphere, and endosphere [21]. Several biomolecules are produced by PGPR, which act as plant growth regulators under saline conditions and allows plants to tolerate the adverse environments. Among these, molecules include the production of phytohormones, the enzyme 1-aminocyclopropane-1-carboxylate deaminase (reduces ethylene levels), siderophores, and microbial exopolysaccharides among others [11,21]. Meanwhile, aluminum toxicity can be diminished by plant-associated bacteria through the production of organic acids, such as malic acid, citric acid, oxalic acid, malonic acid, tartaric acid, and salicylic acid, which not only chelates Al^3+^ but also can bind to phosphorous and reduces the toxic effect of aluminum in roots [22,23,24]. Panhwar et al. [22] also described that exopolysaccharide production could mitigate the aluminum-induced acidification through the absorption of H^+^, increasing pH values at the rhizosphere, and proving a protective layer in the roots, which could chelate metallic ions due to the presence of active functional groups. Aluminum detoxification was described by Mora et al. [25] through the production of siderophores by members of *Klebsiella*, *Stenotrophomonas*, *Enterobacter,* and *Serratia* genera. Iron and aluminum have a similar ionic radius, suggesting the formation of Al^3+^-siderophore complexes and reducing the aluminum toxic effects.

Additionally, Farh et al. [26] suggest the activation of aluminum-stress related genes in *Arabidopsis thaliana* (*AtAIP*: Al-induced protein gene; *AtALS3*: Al-sensitive 3 gene; and *AtALMT1*: Al-activated maleate transporter 1 gene) when plants were treated with PGPR of *Pseudomonas*, *Chryseobacterium*, and *Burkholderia* genera that are isolated from Korean ginseng. The S57 strain has the ability to produce siderophores and IAA to fix elemental nitrogen and to solubilize inorganic phosphate (Table 5); stimulate Micro-Tom tomato growth using irrigation water from Azapa Valley (Table 9 and Figure 5); and can tolerate saline-boric conditions (Table 4); it would be interesting to determine the mechanisms associated to plant-growth promotion for this bacterium and to determine how this strain could increase the plant tolerance to abiotic factors, such as salinity, presence of boron, and aluminum.

The in vitro biocontrol activity of the S57 isolate against phytopathogenic fungi is promising because this property is maintained even under saline-boric conditions (Table 6). Moreover, biocontrol activity against *B. cinerea* was evidenced under the saline-boric conditions of the Azapa Valley using bell pepper plants (Table 10; Figure 6), but no statistical differences relative to control conditions were observed when *F. oxysporum* was used as phytopathogenic fungus. This difference was expected due to the lower in vitro biocontrol activity against *F. oxysporum* determined by the antagonistic assay (Table 6). Additionally, the S57 strain reduced the number of galls formed by *M. incognita* under saline-boric conditions and represents an interesting alternative for developing a new bioproduct with nematicide activity. These results show that the S57 bacterium possesses characteristics of an interesting biocontrol agent with antifungal and nematicide activities which require an exhaustive characterization.

The agricultural potential of the S57 strain renders it an excellent candidate for the development of a new biostimulant and biocontrol agent for agriculture in arid and semi-arid environments. Thus, it is gaining more importance considering that the increasing worldwide soil salinity process could reach 50% of arable soils by 2050 [18] and current commercial bioproducts show non-reproducible results when they are applied to crops maintained under these extreme conditions of cultivation [27].

For the development of bioproducts, it is important to generate a highly concentrated microbial culture for the production’s success. Several factors are crucial for increasing bacterial cell biomass during the production process, including time-saving and cost-effective methods. It is possible to adjust some growth factors, such as pH, temperature, agitation, and incubation time [28], to improve bacterial biomass. For the S57 strain, pH, temperature, and agitation were performed in a flask to determine optimal parameters for inoculum preparation and bacterial scale-up under bioreactor conditions. This bacterium was able to grow optimally at 35 °C in a pH range from 5.5 to 6.0 using 150 rpm of agitation (Table 7) and producing ~12.0 g/L in 48 h, representing a significant increase in biomass in comparison to initial conditions, where less than 1 g/L was produced during 1 week under non-optimal conditions (not showed). Further improvements were achieved when the S57 strain was grown in a fermenter using 0.5 VVM of aeration and 75 rpm of impeller agitation (Table 8), reaching ~47.1 g/L of biomass (~2.5 × 10^27^ CFU/mL) in 24 h.

S57 viability showed to be very stable in the absence and presence of stabilizer compounds (0.1% cocamidopropyl betaine, CMC, Triton X-100, and Tween 20) after six months stored at 4 °C and at room temperature (Figure 2), ranging from ~1 × 10^6^ CFU/mL to ~1 × 10^7^ CFU/mL and increasing their biocontrol activity in vitro against *B. cinerea* (Figure 4) for all formulations. Meanwhile, IAA production (Figure 3) was notoriously increased in 0.1% Triton X-100, making this formulation a potential biostimulant product. Several formulations based on *Pseudomonas* bacteria have been reported. For *Pseudomonas fluorescens* AMB-8 where a notorious decrease in viable cell counting was detected when the bacterium was incubated at room temperature for 6 months without the addition of any protective agent to King’s medium B or nutrient broth. The amendment of nutrient broth with 2% glycerol improved the viability of this strain to 10^7^ CFU/mL compared to 10^5^ CFU/mL in non-amended nutrient broth [29]. He et al. [30] also observed an improvement in the viability of *Pseudomonas putida* RS-198 when bentonite, alginate, CMC, and polyvinyl alcohol were used as stabilizer agents to culture medium after 6 months of incubation. Particularly for the S57 strain, non-notorious improvements in viability were observed when protective agents were added to the bacterial formulation. A bacterium-based bioproduct must be formulated appropriately to guarantee that it will provide all the beneficial traits that it is supposed to afford. The incorporation of stabilizers substances to S57 liquid formulation allowed maintaining higher viable cells and functionally active bacteria during long-term storage. Several protective compounds have been employed to increased bacterial viability during time, including natural polymers (carrageenan, arabic gum, xanthan gum, gelatin, and alginate), synthetic polymers (polyvinyl alcohol and polyvinylpyrrolidone), horticultural oils, glycerol, and mono-saccharides and di-saccharides; all of these must protect the bacterium from abiotic stress generated during storage [31]. It is important to mention that solid formulations (granules and dry and wet powders) could extend the S57 strain stability over time. For example, *Pseudomonas tolaasii* IEXb maintained its viability after 6 months stored at 4 °C, when it was freeze-dried using whey as the carrier and sodium glutamate as the stabilizer agent; meanwhile, a minor reduction in viable bacterial cells from 10^9^ CFU/mL to 10^6^ CFU/mL was observed at room temperature [31]. The saline-boric tolerance, PGP traits, and biocontrol activities for the S57 strain are promising and reveal the possibility to develop a new biostimulant and biofungicide that are functional and active under arid and semi-arid conditions. Additionally, the produced biomass of the S57 strain is attractive compared to other bacterial products and allow high quantities of the bacterium to be obtained in a single fermentation step. However, further studies are necessary to improve the viability during storage and to characterize the mechanisms of plant-growth promotion and antifungal biocontrol activity.

## 4. Materials and Methods

### 4.1. Sampling and Bacterial Isolation

Sample collection was performed at three sites in Socoroma, Arica and Parinacota Region, Chile, in 2017 (Table 1). Samples consisting of entire oregano plants were collected aseptically using a metallic shovel disinfected with 70% (*v*/*v*) ethanol and stored in sterilized plastic bags (17 × 11 inch). Soil samples were taken for composition and pesticide residues analysis (Analab, Santiago, Metropolitan Region, Chile). Samples were kept at 4 °C in a cooler and immediately transported to the laboratory for processing.

The S57 strain was isolated following the procedure described by Muñoz et al. [6] and is part of the plant-associated bacterial culture collection of the Laboratory of Plant Pathology and Bioproducts belonging to the Universidad de Tarapacá. This collection comprises one hundred eighty bacteria, which is functionally characterized for the bacterial PGP traits. The S57 isolate was selected from the culture collection due to their promising in vitro PGP activities and obtained using the method described by Yang et al. [32] by cutting oregano roots into small parts and disinfected using 95% (*v*/*v*) ethanol for 2 min, 2% (*v*/*v*) sodium hypochlorite for 2 min, and 70% (*v*/*v*) ethanol for 2 min followed by two washes of autoclaved distilled water for 2 min each time. The disinfected portions were placed into plates of King’s medium B [33] containing the following (per liter): 20.0 g peptone, 10.0 mL glycerol, 1.5 g K_2_HPO_4_, 1.5 g MgSO_4_·7H_2_O, and 15.0 g agar (pH 7.0); and incubated at 25 °C for 1 week or until microbial growths were observed. Colonies were isolated using serial dilutions on King’s B broth and streaking on plates of solid medium. Incubations were performed at room temperature. Isolated colonies were transferred to a liquid medium. These procedures were repeated until a single and homogeneous morphology was observed under the microscope.

The isolated S57 strain was deposited in the Chilean Collection of Microbial Genetics Resources under the accession number RGM2930.

### 4.2. Bacterial Identification

Genomic DNA from selected PGPR was obtained using the DNeasy UltraClean Microbial kit (QIAGEN, Germantown, Maryland, USA) according to the procedure described by the manufacturer. The 16S rRNA gene was amplified by PCR using bacteria-specific primers 27F and 1492R [34]. PCR reaction mix and PCR cycles were performed according to the procedure described by Muñoz et al. [6]. Amplification reactions were performed using a VeritiTM 96-well Thermal Cycler (Thermo Fisher Scientific, Waltham, Massachusetts, USA). A band of ~1500 bp was observed after amplification on 1.0% (*w*/*v*) agarose gel prepared in 1X TAE buffer (40 mM Tris-acetate, 10 mM EDTA) and visualized under UV light using 1X GelRed (Biotium (San Francisco, LA, USA). The PCR products were sequenced using the primers described above (Macrogen (Seoul, Korea)) and manually edited using the ChromasPro software (http://technelysium.com.au/wp/chromaspro/ accessed on 1 April 2020) to remove low-quality bases. Forward and reverse sequences were assembled using the Megamerger tool (http://www.bioinformatics.nl/cgi-bin/emboss/megamerger accessed on 1 April 2020) to obtain a sequence length of 1448 bp. The partial sequence was compared to GenBank using BLAST software [35]. The partial 16S rRNA gene sequence was deposited in the GenBank nucleotide sequences databank under accession number MK883138.

The partial sequence of 16S rRNA obtained from the S57 strain and selected sequences belonging to the *Pseudomonas* genus retrieved from GenBank were aligned using the Clustal W software [36]. The alignment was manually edited to obtain sequences of similar length. Phylogenetic analysis was carried out using the MEGA7 software [37] and by considering the *Aquifex pyrophilus* strain Kol5a as the outgroup. The phylogenetic tree was inferred from the multiple sequence alignments by using the Neighbor-Joining method and by using a bootstrap analysis of 1000 replicates to determine the reliabilities of each node.

### 4.3. Bacterial Characterization

The phenotypic characterization of the S57 strain was performed according to the procedure described by Muñoz et al. [38].

Antibiotic susceptibility was carried out using the disk diffusion method according to Simirgiotis et al. [39] by using different antibiotics as observed in Table 3. Two dissimilar quantities of each antibiotic were employed to determine if the S57 strain had differences in its behavior in the susceptibility test. The assay was performed using five independent replicates.

### 4.4. Tolerance of the S57 Strain to NaCl and H_3_BO_3_

The tolerance of the S57 strain to NaCl was determined using King’s medium B supplemented with 0–20 g/L of NaCl. The tolerance to H_3_BO_3_ was determined using King’s medium B amended with 0–100 ppm of H_3_BO_3_.

A mixture consisting of King’s medium B supplemented with 0.86 g/L NaCl and 114 ppm of H_3_BO_3_ (1×) was used to emulate the characteristics of the irrigation water of the Lluta River, which is the main source of water for crops in Socoroma [40], and to determine the tolerance of the S57 strain proving that this bacterium can grow under the extreme water conditions of this zone. Furthermore, the medium was amended with a higher concentration up to 10× the irrigation water of the Lluta River.

Bacterial growth was monitored each day during one weak of incubation through the measurement of OD_600_ using a spectrophotometer. Bacterial growth was registered as ++ for abundant growth, which is similar to control condition (absent of NaCl and H_3_BO_3_); + for poor growth; and – for no growth. All assays were carried out using three independent replicates.

### 4.5. In Vitro PGP Traits

Inorganic phosphate solubilization was detected in Pikovskaya (PVK) solid medium according to the method described by Pikovskaya [41]. The appearance of a clearing zone around the bacterial colony after incubation is indicative of phosphate solubilization.

Nitrogen fixation was determined using NFb semisolid medium using the procedure described by Rodríguez [42] in which nitrogen fixation is defined by the formation of a sub-superficial whitish ‘veil-like’ pellicle after incubation.

The production of IAA was quantified using the colorimetric Salkowski’s method [43]. Bacterial supernatant was mixed with Salkowski’s reagent (0.5 M FeCl_3_ in 35% HClO_4_) in the ratio of 1:2 (supernatant: Salkowski’s reagent) in the dark at room temperature. A calibration curve was adjusted using an IAA standard using the concentration range 0–50 μg/mL and the absorbance at 530 nm was measured using a T60 UV/VIS spectrophotometer (PG Instruments Limited).

Siderophores production was quantified by the Chrome Azurol S (CAS) method described by Schwynand and Neilands [44], where a ratio of 1:1 of supernatant: CAS reagent was employed and the absorbance at 630 nm was registered using a UV/VIS spectrophotometer after 20 min of incubation. Siderophore production was in percent siderophore unit (psu) according to the following formula:

psu = (Ar − As) × 100/Ar

where Ar is the absorbance of the reference (CAS solution and un-inoculated medium) and As is the absorbance of the supernatant of the S57 strain.

All experiments for in vitro PGP traits were carried out using three independent replicates.

### 4.6. Antifungal Activity

In vitro growth inhibition of phytopathogenic fungi was determined through a dual culture assay performed in potato dextrose agar (PDA) plates by using the phytopathogenic fungi *B. cinerea*, *F. oxysporum*, *G. candidum*, and *M. fructicola* inoculated at the center of each plate and aliquots of the S57 strain were inoculated surrounding the fungus according to the method described by Sepúlveda-Chavera et al. [45]. As controls, plates with only the fungus in the center were used. The inhibition of the mycelial radial growth (IMRG) of the fungus will be calculated using the following equation:

IMRG = [(C − T)/C] × 100

where C is the growth area of the fungus in the control plate and T is the fungal growth area using the treatment.

Moreover, the same experiment was performed using PDA plates amended with 10 g/L NaCl and 110 ppm H_3_BO_3_ (saline-boric conditions) to determine if biocontrol activity against phytopathogenic fungi is maintained under saline-boric conditions. Furthermore, dual culture assays against *B. cinerea* and *F. oxysporum* (common phytopathogenic fungi in the Arica and Parinacota Region) were also performed using PDA plates prepared with twice-autoclaved irrigation water from the Lluta River instead of distilled water.

All antifungal assays were carried out using three independent replicates.

### 4.7. S57 Culture Optimization

A growth curve was performed by taking 1 mL aliquots each 1 h during 48 h from the culture medium inoculated with the S57 strain. Bacterial growth was monitored by the increase in OD_600_ using a spectrophotometer and viable count was monitored busing serial dilutions on a solid medium.

Flask-experiments were conducted until the stationary phase using a final volume of 200 mL of bacterial culture. This strategy was used to characterize and model the behavior of the S57 strain by defining the variables of the process (agitation, pH, and temperature) to promote bacterial growth in the flask. The effect of each parameter was recorded by obtaining growth curves and calculating generational time (g) and microbial growth rate (μ). Two variables were maintained constant to determine each optimum parameter.

The S57 strain was inoculated in King’s medium B and the temperature was maintained in a shaker incubator to determine the temperature effect. The range of temperature to be assayed was 25–40 °C.

The S57 strain was inoculated in King’s medium B and pH was buffered to 5.0–6.0 using 50 mM MES and 6.5–7.5 using 50 mM HEPES in order to determine the pH effect and adjusted to their respective values using NaOH or HCl.

The effect of agitation was determined in a shaker incubator at room temperature by using different agitation speeds between 0–150 rpm.

The S57 strain culture was scaled up in a one-liter bioreactor (BioFlo^®^/CelliGen^®^ 115 fermenter) under optimal flask-conditions. Optimization in the bioreactor was performed by using the design of a two-variables experiment where aeration and agitation were modified simultaneously according to Table 13. The experimental temperature was maintained at 35°C and pH was controlled to 5.5–6.0 using 5% (*w*/*v*) H_3_PO_4_ and 5% (*w*/*v*) NaOH. Biomass and viable count were determined to select optimal growth conditions of the S57 strain in the bioreactor.

For viable counts, samples of 1 mL were taken after 24 h of incubation and serial dilutions were prepared in sterile King’s medium B. Each dilution was inoculated in a solid medium and colonies counting was performed after incubation to determine colony-forming units per mL (CFU/mL).

Furthermore, bacterial viability was determined once a month using dilutions of the S57 strain to ~5 × 10^9^ CFU/mL and stored at room temperature and 4 °C for six months. For this purpose, four formulations were generated using the stabilizing agents carboxymethylcellulose (CMC), cocamidopropyl betaine, Tween 20, and Triton X-100 at 0.1% final concentration.

Samples of 1 mL were taken once a month and serial dilutions in sterile King’s medium B were performed. Each dilution was inoculated in a solid medium and colony counting was determined after incubation as described above. Experiments were executed using three replicates. Moreover, in vitro antifungal activities against *B. cinerea* and auxin production were monitored monthly according to the procedure described above.

### 4.8. PGP Activity of the Strain S57 in Micro-Tom Tomato Plants

PGP activity was also evaluated in Micro-Tom tomato plants (Tomato Genetics Resource Center, University of California Davis). Micro-Tom tomato seeds were disinfected using 95% (*v*/*v*) ethanol for 2 min, 2% (*v*/*v*) sodium hypochlorite for 2 min, and 70% (*v*/*v*) ethanol for 2 min followed by two washes with sterile distilled water for 2 min each time. Seeds were germinated in a sterile dark wet chamber at 25 °C for ten days. After their germination, Micro-Tom seedlings were transferred to pots containing twice autoclaved perlite as the only substrate and were kept in a greenhouse. Plants were treated once a week for a month using 1 × 10^8^ CFU of the S57 strain by the application of the bacterium at the stem’s base. The fresh inoculum was prepared and diluted in sterile water. Micro-Tom tomato plants treated only with sterile King’s medium B were used as a control. Plants were watered daily with irrigation water from the Azapa Valley. After 4 weeks of inoculation, the Micro-Tom plants were removed from the substrate and washed and the length of the stem, wet weight, and dry weight of the roots and the aerial parts were measured separately. Experiments were carried out using five independent replicates.

### 4.9. Biocontrol Activity of the S57 Strain in Planta Against Phytopathogenic Fungi

In order to determine the biocontroller effect of the S57 bacterium, a planta assay was designed in pots with plants of bell pepper cv. Almuden (Syngenta^®^). The plants were obtained from a commercial nursery and transplanted when they had the first true expanded leaf (two weeks of germination). The experimental design corresponded to a completely randomized design with uniform management. The application of bacteria suspended in water was the only source of variation. A positive control treatment (Gold Standard, GS) corresponding to *Bacillus subtilis* (Serenade^®^-Syngenta) was considered. Plants that were not inoculated and treated only with water were used as a negative control. The plants were grown in commercial peat as substrate and fertilized after the first week of transplantation and watered daily. The experimental design considered three replicates. After 10 days of transplantation, two phytopathogenic fungi were incorporated: *B. cinerea* and *F. oxysporum*. For the inoculation of bell pepper plants, 2 mL of a suspension (10^6^ CFU/mL) of *B. cinerea* conidia was applied to each plant. *F. oxysporum* was inoculated in conidial suspension (10^6^ CFU/mL) by applying 2 mL per plant at a location close to the neck of the plants. Each fungus was applied in independent pots.

The S57 strain and the GS were applied three times: 5, 15, and 25 days after transplantation. The development of foliar symptoms and plant height were evaluated. For each application, an independent suspension of each bacterium was prepared in a liquid proteose peptone medium and it was cultivated at 35 °C for 48 h using 150 rpm of shaking (obtaining a concentration of 1 × 10^9^ CFU/mL). For the application in plants, 1 mL of the medium with bacterial growth was taken and it was suspended at a rate of 1 mL of broth in 1 L of sterile distilled water. Three mL of bacterial suspension were applied to each plant using a manual spray pump.

### 4.10. Nematicide Activity of the S57 Strain in Planta

#### 4.10.1. Nematode Isolation

The nematode *M. incognita* was obtained from an axenic population maintained in tomato plants cv. *Poncho Negro*. The nematode collection was performed by the method described by Hussey and Barker [46] and modified by Bonetti and Ferraz [47]. The roots of tomato plants infested by the nematode were gently washed to release the adhered soil and macerated in the presence of 0.5% NaClO. The nematode suspension was filtered using a 500-mesh sieve to retain the eggs; the total count of the eggs was conducted in a Peters chamber using a stereomicroscope. The suspension was calibrated to 5000 eggs/mL.

#### 4.10.2. In Planta Nematicide Assay

In order to determine the nematicide effect of the S57 strain, tomato plants cv. *Poncho Negro* were transplanted to 3 L pots using a mixture of peat: perlite (2:1) as soil substrate. Four treatments were considered for the assay: tomato plants without *M. incognita* eggs (Control without inoculating), tomato plants inoculated only with *M. incognita* eggs (Control without controller), tomato plants inoculated with *M. incognita* eggs and treated with fluopyram at commercial dosses (3 mg/plant diluted in irrigation water) (Chemical control), and tomato plants inoculated with *M. incognita* eggs and treated with the S57 strain. After two days of transplantation, one mL of *M. incognita* eggs (~5000 eggs/mL) was inoculated to each respective plant in the roots. For the S57 strain, one mL of bacterial suspension (1 × 10^6^ CFU/mL) was added twice with ten days of separation. Tomato plants were cultivated for 35 days. The experiment represented a completely randomized design with 4 replicates. After cultivation, 100 g of roots were obtained and the number of galls was determined for each treatment.

## 5. Conclusions

The S57 strain is an endophytic bacterium associated with oregano roots from Socoroma (the Atacama Desert) and it is a member of the *Pseudomonas* genus that is closely related to *P. lini*. This bacterium has PGP traits, including the ability to fix nitrogen, solubilize phosphorous, produce IAA and siderophores, and promote the growth of aerial parts of Micro-Tom tomato plants. Furthermore, the S57 strain can tolerate saline boric conditions. It can inhibit the mycelial growth of phytopathogenic fungi in vitro, even in the presence of NaCl and H_3_BO_3_ which renders it a good candidate for the development of a new bioproduct functional and active under desertic conditions. Moreover, the S57 bacterium possesses biocontroller activity in planta against the fungus *B. cinerea* and the nematode *M. incognita* under saline-boric conditions. This bacterium can be produced in high quantities and remains viable and functionally active after 6 months of storage. However, it is necessary to improve its viability over time and further characterize the mechanisms associated with PGP and biocontrol activities.

## 6. Patents

Two patent applications were requested to INAPI under the accession number 202000346 and 202000348.

## Figures and Tables

**Table 1:** Composition of soil samples from Socoroma.

Parameter	S1	S2	S3
GPS Location	18°15′13.3″ S 69°36′36.0″ W	18°15′14.8″ S 69°36′46.3″ W	18°15′50.1″ S 69°35′°39.0″ W
Altitude (m.a.s.l)	2909	2892	3090
Total N (%)	0.37	0.20	0.07
P (mg/kg)	77.30	57.00	21.49
K (mg/kg)	0.85	0.79	0.77
S (mg/kg)	24.00	44.50	16.00
B (mg/kg)	13.00	0.68	0.04
Al (cmol/kg)	11.90	5.12	0.12
Cu (mg/kg)	4.11	3.70	0.80
Na (cmol/kg)	0.28	0.33	0.30
pH	4,66	4.75	6.11
Organic matter (%)	4.87	2.58	0.79
Electrical conductivity (dS/cm)	87.6	116.2	102.2

**Table 2:** Microbiological characterization of the S57 strain. A positive reaction is indicated as +; meanwhile, no reaction is indicated as −.

Parameter	S57
Gram staining	−
Morphology	Straight rods
Motility test	+
Catalase	+
Acid production from:	
D-Sorbitol	−
D-Mannitol	+
D-Glucose	+
D-Fructose	+
D-Galactose	+
D-Rhamnose	+
D-Melibiose	+
D-Cellobiose	−
Sucrose	+
Lactose	+

**Table 3:** Antibiotic susceptibility test of the S57 strain. Two antibiotic quantities were employed to determine differences in the behavior of S57 bacterium in the susceptibility test. Error corresponds to the standard deviation of the independent assays.

Antibiotic	Quantity	Inhibition Diameter (cm)	Susceptibility
Amoxicillin	2 μg	0.0	Resistant
	25 μg	0.0	Resistant
Ampicillin	2 μg	0.0	Resistant
	25 μg	0.0	Resistant
Chloramphenicol	10 μg	12.3 ± 0.4	Intermediate
	50 μg	24.3 ± 1.0	Sensitive
Ciprofloxacin	1 μg	32.0 ± 0.5	Sensitive
	10 μg	40.7 ± 0.3	Sensitive
Kanamycin	5 μg	15.0 ± 1.0	Intermediate
	30 μg	22.3 ± 0.4	Sensitive
Neomycin	10 μg	10.3 ± 0.4	Intermediate
	30 μg	14.0 ± 0.0	Intermediate
Penicillin G	1 U	0.0	Resistant
	10 U	0.0	Resistant

**Table 4:** Tolerance of the S57 strain to NaCl and H_3_BO_3_**.** Bacterial growth was registered as ++: abundant growth, similar to control condition, and in the absence of NaCl and H_3_BO_3_; +: poor growth; and −: no growth.

Condition	Bacterial Growth
Control	++
8 g/L NaCl	++
15 g/L NaCl	++
20 g/L NaCl	+
10 ppm H_3_BO_3_	++
50 ppm H_3_BO_3_	++
100 ppm H_3_BO_3_	++
1× Lluta irrigation water	++
9× Lluta irrigation water	+
10× Lluta irrigation water	−

**Table 5:** In vitro PGP traits of the S57 strain. A positive reaction is indicated as +. Error corresponds to standard deviation of the independent assays.

PGP Trait	Result
Nitrogen fixation	+
Phosphate solubilization	+
(IAA) (μg/mL)	7.9 ± 0.5
Siderophore production (psu)	24.2 ± 3.16

**Table 6:** In vitro antifungal activity of the S57 strain. Results are indicated as percentage of IMRG. Error corresponds to standard deviation of three independent assays. N.D.: not determined.

Fungus	IMRG (%)		
	Standard Conditions	Saline-Boric Conditions	Lluta Irrigation Water
*B. cinerea*	48.3 ± 5.8	42.8 ± 5.3	43.3 ± 4.4
*F. oxysporum*	23.5 ± 2.8	26.3 ± 3.2	22.8 ± 4.2
*G. candidum*	58.6 ± 7.0	29.6 ± 3.6	N.D.
*M. fructicola*	67.2 ± 8.1	52.3 ± 6.3	N.D.

**Table 7:** Optimal conditions for the growth of S57 bacterium. Each optimum parameter was determined by maintaining the other two parameters invariable. Error corresponds to the standard deviation of three independent assays.

Parameter	g (h)	μ (h^−1^)
Temperature (°C)		
25	0.35 ± 0.03	2.85 ± 0.24
30	0.34 ± 0.04	2.89 ± 0.34
35	0.33 ± 0.02	2.98 ± 0.18
40	1.26 ± 0.22	0.79 ± 0.14
pH		
5.0	0.48 ± 0.04	2.07 ± 0.17
5.5	0.30 ± 0.02	3.45 ± 0.23
6.0	0.30 ± 0.01	3.32 ± 0.11
6.5	0.31 ± 0.03	3.25 ± 0.31
7.0	0.32 ± 0.03	3.13 ± 0.29
7.5	0.31 ± 0.04	3.25 ± 0.42
Agitation (rpm)		
0	6.54 ± 0.50	0.15 ± 0.01
50	1.89 ± 0.17	0.53 ± 0.05
100	0.46 ± 0.09	2.16 ± 0.42
150	0.33 ± 0.04	3.06 ± 0.37

**Table 8:** Optimization of growth of the S57 strain in bioreactor. Error represents standard deviation of the independent assays.

Experiment Number	Log (CFU/mL)	Biomass (g/L)
1	23.2	35.9 ± 5.40
2	21.3	33.0 ± 6.60
3	19.0	29.4 ± 7.35
4	22.1	34.5 ± 6.90
5	23.9	37.1 ± 4.50
6	27.4	47.1 ± 6.30
7	26.9	41.7 ± 6.30

**Table 9:** Effect of the application of the S57 strain in the Micro-Tom tomato plants growth. Error represents standard deviation of the five independent assays.

	Control	S57 Treatment	Fold
Stem length (cm)	11.8 ± 1.40	15.7 ± 1.10	1.33
Root wet weight (g)	2.3 ± 0.40	2.7 ± 0.50	1.18
Root dry weight (g)	0.18 ± 0.04	0.18 ± 0.04	1.00
Aerial wet weight (g)	2.36 ± 1.00	5.35 ± 1.00	2.27
Aerial dry weight (g)	0.57 ± 0.10	0.83 ± 0.10	1.46

**Table 10:** Height of bell pepper plants cv. Almuden treated with S57 strain in comparison to control and GS treatments. Error represents the standard deviation of the three independent assays. The same letters represent statistical equality (Tukey test, *p* ≤ 0.05).

Treatment	*B. cinerea*	*F. oxysporum*
S57	26.0 ± 1.0 ^b^	26.0 ± 1.0 ^A^
Serenade	21.7 ± 1.5 ^a^	26.0 ± 1.0 ^A^
Control	23.0 ± 1.0 ^a^	27.7 ± 2.1 ^B^

**Table 11:** Fresh weight of the third leaf of pepper plants cv. Almuden treated with the S57 strain in comparison to control and GS treatments. Error represents the standard deviation of the four independent assays. The same letters represent statistical equality (Tukey test, *p* ≤ 0.05).

Treatment	Fresh Weight (g)
S57	1.68 ± 0.3 ^b^
Serenade	1.34 ± 0.3 ^a^
Control	1.66 ± 0.5 ^b^

**Table 12:** The number of galls of *M. incognita* per 100 g of roots of tomato plants cv. *Poncho Negro*. Error represents standard deviation of the four independent assays. The same letters represent the statistical equality (Tukey test, *p* ≤ 0.05).

Treatment	N° Galls
Control without inoculate	0 ± 0 ^a^
Control without controller	2428 ± 109 ^b^
S57	689 ± 83 ^c^
Fluopyram	2 ± 2 ^a^

**Table 13:** Experimental design to optimize S57 strain culture conditions in bioreactor.

Experiment Number	Aeration (VVM)	Agitation (rpm)
1	0.5	100
2	1.0	100
3	1.5	100
4	0.5	50
5	1.5	50
6	0.5	75
7	1.5	75
8	0.5	150
9	1.0	150
10	1.5	150

## Data Availability

Data is contained within the article.
